# Ankyrin-mediated self-protection during cell invasion by the bacterial predator *Bdellovibrio bacteriovorus*

**DOI:** 10.1038/ncomms9884

**Published:** 2015-12-02

**Authors:** Carey Lambert, Ian T. Cadby, Rob Till, Nhat Khai Bui, Thomas R. Lerner, William S. Hughes, David J. Lee, Luke J. Alderwick, Waldemar Vollmer, Elizabeth R. Sockett, Andrew L. Lovering

**Affiliations:** 1 Centre for Genetics and Genomics, School of Biology, Nottingham University, Medical School, Queen's Medical Centre, Nottingham NG7 2UH, UK; 2 Institute for Microbiology and Infection, School of Biosciences, University of Birmingham, Birmingham B15 2TT, UK; 3 Centre for Bacterial Cell Biology, Institute for Cell and Molecular Biosciences, Newcastle University, Richardson Road, Newcastle upon Tyne NE2 4AX, UK

## Abstract

Predatory *Bdellovibrio bacteriovorus* are natural antimicrobial organisms, killing other bacteria by whole-cell invasion. Self-protection against prey-metabolizing enzymes is important for the evolution of predation. Initial prey entry involves the predator's peptidoglycan DD-endopeptidases, which decrosslink cell walls and prevent wasteful entry by a second predator. Here we identify and characterize a self-protection protein from *B. bacteriovorus*, Bd3460, which displays an ankyrin-based fold common to intracellular pathogens of eukaryotes. Co-crystal structures reveal Bd3460 complexation of dual targets, binding a conserved epitope of each of the Bd3459 and Bd0816 endopeptidases. Complexation inhibits endopeptidase activity and cell wall decrosslinking *in vitro*. Self-protection is vital — ΔBd3460 *Bdellovibrio* deleteriously decrosslink self-peptidoglycan upon invasion, adopt a round morphology, and lose predatory capacity and cellular integrity. Our analysis provides the first mechanistic examination of self-protection in *Bdellovibrio*, documents protection-multiplicity for products of two different genomic loci, and reveals an important evolutionary adaptation to an invasive predatory bacterial lifestyle.

## Abstract (web summary)

The bacterium *Bdellovibrio bacteriovorus* invades and kills other bacteria, but it is unclear how it avoids degradation of its own cell wall. Here the authors identify the *B. bacteriovorus* protein Bd3460 as an endopeptidase inhibitor that prevents hydrolysis of the predator's peptidoglycan during invasion of prey.

## Graphical abstract

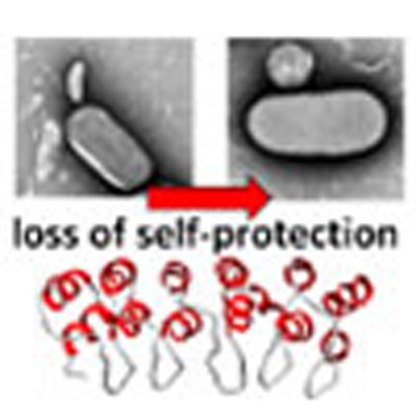

B*dellovibrio* are deltaproteobacteria that enter and kill diverse pathogenic Gram-negative bacterial species and have been tested as possible whole-cell antibacterial agents^1^,^2^. *Bdellovibrio bacteriovorus* is a periplasmic predator that enters through the outer membrane of prey and metabolizes the ‘infected' cell from within^3^. The intracellular lifestyle of *B. bacteriovorus* requires many specialized adaptations^4^, one of which is the formation of the osmotically-stable bdelloplast, wherein the (usually) rod-shaped prey cell becomes rounded up immediately after *Bdellovibrio* instigates prey invasion^5^. This rounding is caused by prey peptidoglycan cell wall modification, catalysed by *Bdellovibrio* enzymes^6^. The *B. bacteriovorus* genome encodes many predation-associated genes, additional to those for self cell wall maintenance. These gene products modify prey peptidoglycan in sequential, but different, ways to facilitate bdelloplast formation: withstanding initial predator-invasion without breaking; accommodating the invasive *Bdellovibrio* growing within; withstanding and becoming ready for final lysis when *Bdellovibrio* replication is complete. Although these cell cycle concepts and associated cell wall modifications are predicted and seen microscopically as events, few activities have been directly attributed to specific *Bdellovibrio* predatory gene products. Previously, we assigned prey cell rounding to the action of two secreted *Bdellovibrio* peptidoglycan DD-endopeptidases, Bd0816 and Bd3459, that act to modify the invaded cell wall via hydrolysis of the structural 3-4 peptide crosslinks^6^. The prey morphology-change catalysed by these enzymes functioned as an ‘occupancy signal', preventing wasteful entry by successive predators and speeding up prey invasion^6^. Thus, rounded prey-bdelloplast formation, catalysed by the pair of DD-endopeptidase enzymes (Bd3459, Bd0816) was found to promote a 1:1 predator to prey cell ratio and drive population fitness, preventing self-competition between individual *Bdellovibrio* for the same prey cell. This feature is important as although the long range encounter between predator *Bdellovibrio* and a cloud of potential prey involves chemotaxis^7^; the final short range encounter cannot be guided by chemotaxis as bacteria do not sense chemotactically along their cell bodies at short range^8^. Thus, several *Bdellovibrio* will arrive at a single prey cell as they cannot use self or prey sensing to prevent this. Furthermore, *Bdellovibrio* are released as a cloud of predators by lysis of an adjacent infected bdelloplast. However, the ‘occupancy signal' of DD-endopeptidase-mediated rounding does prevent multiple entry to a single prey cell. Thus these DD-endopeptidase enzymes are vital to predation efficiency but they also target peptidoglycan, which is common to both predator and prey; the evolutionary fitness benefit of eliminating auto-competition brings with it a risk of self-damage which must be mitigated.

Structure and activity analyses of DD-endopeptidase Bd3459 revealed a highly active enzyme with an open active site adapted for prey peptidoglycan diversity, rather than self-wall maintenance (this was also inferred for the homologous Bd0816). This raised the intriguing question of how *Bdellovibrio* protects its own cell wall from modification/destruction by Bd3459 and Bd0816 passing through its own periplasm when invading prey^6^. To this end, we instigated a search to find a potential ‘self-protection protein' that could act to block endopeptidase activity in the predator, while such DD-endopeptidases were being secreted, past their own peptidoglycan cell wall to that of the prey.

Here we show that *Bdellovibrio bacteriovorus* utilizes a small ankyrin repeat protein, Bd3460, to protect itself from endopeptidase activities during entry of prey. We demonstrate that endopeptidase complexation by Bd3460 prevents cell wall decrosslinking, and that both the Bd0816 and Bd3459 targets bind this self-protection protein via a common epitope. Predators lacking this protection are observed to deleteriously self-round upon cell contact and endopeptidase induction; thus forming an abortive ‘spheroplast-like shape' at the entry pore and negating prey cell entry & killing.

## Results

### Characterization of a *Bdellovibrio* self-protection protein

Reasoning that protective protein(s) should act on both DD-endopeptidase gene products in the periplasm, we examined the gene neighbourhoods of *bd0816* and of *bd3460* initially looking for a common pair of potential ‘immunity genes', which we didn't find. Gene *bd0816* is preceded by a small 150 bp gene without a signal peptide; *bd3460* however encodes a small 23 kDa protein, with predicted ankyrin repeats on a signal peptide. Ankyrin-repeat proteins (ARPs) are often involved in protein-protein interactions and can be found in several toxins and their associated immunity proteins^9^,^10^. Interestingly, ARPs are rare in bacteria, but are enriched in intracellular parasites of eukaryotes where they are chiefly used to modulate host cell processes^11^. The *bd3459* endopeptidase/*bd3460* ARP gene synteny, albeit with ARP expressed after the protein it should protect against (see explanation later), is shared in other periplasmic predator genomes^12^, and is absent in related strains exhibiting epibiotic predation (which adhere to prey but do not invade^13^). This cumulative evidence suggested that Bd3460 could represent the first self-protection protein identified in predatory bacteria, a hypothesis we test and validate in the present study.

Co-transcription of *bd3459* and *bd3460* was established via semi-quantitative RT-PCR (Supplementary Fig. 1, showing peak expression at prey invasion timepoints). Co-purification of tagged Bd3459/Bd0816 and untagged Bd3460 indicated a 1:1 complex formation *in-vitro*. These interactions were confirmed and quantified using intrinsic tryptophan fluorescence emission measurements (Supplementary Fig. 2); with an estimated affinity of Bd3460 for Bd3459 of 26.8 uM. Acylation of the DD-endopeptidase active site serine by the specific inhibitor penicillin G caused an approximate 2-fold reduction in affinity of Bd3460 for Bd3459.

We utilized an identical assay to our original Bd3459 characterization^6^, wherein isolated peptidoglycan from a pentapeptide-rich strain of *Esherichia coli* is incubated with enzyme and modifications are monitored via endpoint HPLC analyses. Purified recombinant Bd3460 completely inhibited the endopeptidase activity of Bd3459, as expected for a functional immunity protein (Supplementary Fig. 3).

**Figure 1:**
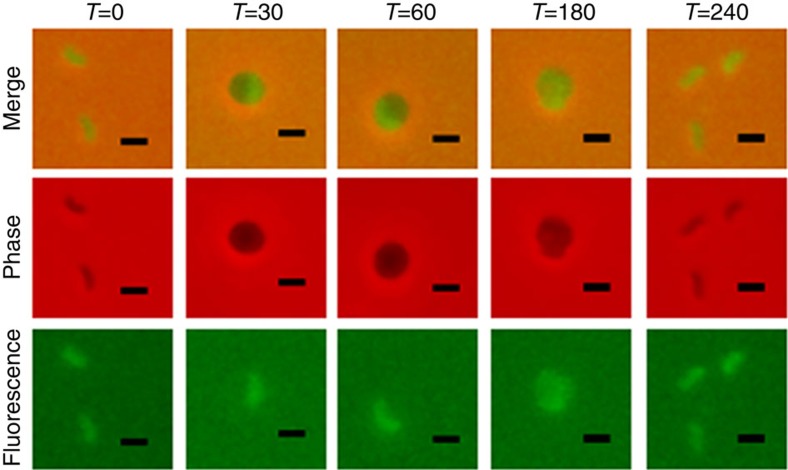
Periplasmic localization of Bd3460 protein. Epifluorescence phase contrast microscopy of *Bdellovibrio* with a Bd3460::mCherry tag. Fluorescence is seen in the small, attack phase cells at times 0 and 240 min, and increases as the *Bdellovibrio* enter the prey, which rounds up to form a ‘bdelloplast'. As the *Bdellovibrio* cell grows inside the bdelloplast, the fluorescence becomes dissipated in the larger, cylindrical cell (*T*=180 min). Scale bar, 1 μm.

Gene *bd3460* lies downstream of *bd3459* raising the questions: how does it protect the cell when *bd3459* is transcribed, and translated before it; and how may Bd3460 protect against Bd0816 transcribed and translated from a gene locus elsewhere on the genome? Monitoring fluorescence of Bd3460::mCherry throughout the predatory cycle showed that Bd3460 is expressed in pre-invasive *Bdellovibrio* (Fig. 1), and expression increased slightly during *Bdellovibrio* prey-entry and rounding (at the time that Bd3459 and Bd0816 are utilized to effect prey cell wall decrosslinking). Released, daughter attack-phase *Bdellovibrio* maintained Bd3460::mCherry fluorescence, indicating that constant Bd3460 availability is the mechanism by which the *Bdellovibrio* cell is protected from the upstream, earlier expressed Bd3459 and Bd0816 upon the next prey encounter and invasion.

### Prey-expression of Bd3460 protects against decrosslinking

**Figure 2:**
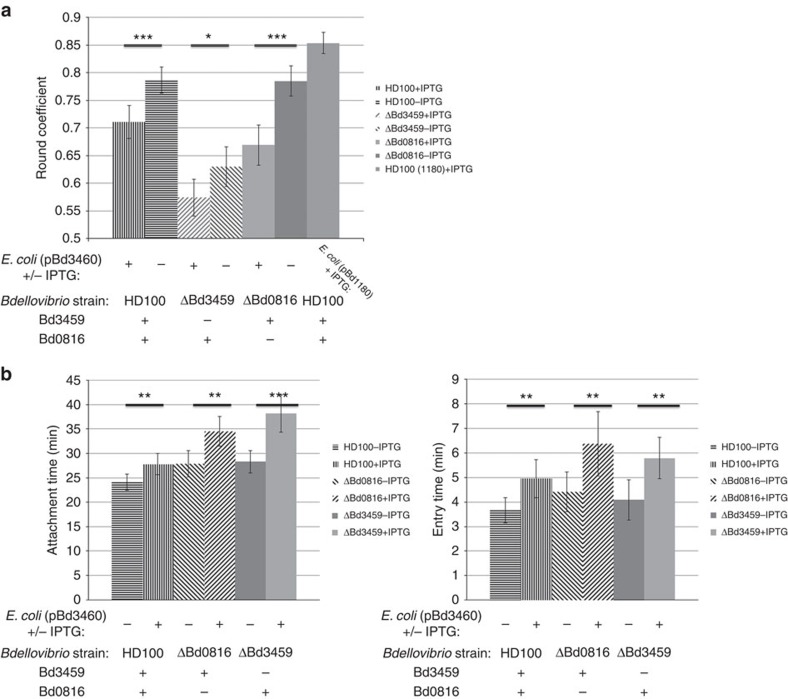
Heterologous Bd3460 protects prey from rounding and affects predator entry. (**a**) Graphs showing the average roundness coefficient of bdelloplasts from phase contrast images. Roundness analysis was carried out on wild-type and endopeptidase knockout-mutant *Bdellovibrio* infected *E. coli* prey cells heterologously expressing Bd3460. Images were taken 90 min post-invasion and the roundness of infected prey cells were analysed using ImageJ software. Roundness of bdelloplasts is reduced in all cases by IPTG induction of *bd3460*. A negative control of induction of *bd1180*, a different ankyrin repeat protein, did not reduce roundness, giving values similar to wild type (0.85 c.f. 0.90 published in Lerner *et al*.^6^) Error bars show 95% confidence intervals and statistical analysis of the means were compared with WT (*P<0.05; ***P<0.001 as determined by Student's *t*-test). Data are taken from at least two independent experiments (*n*>60). (**b**) Histograms of mean times for attachment (lefthand panel) and invasion (righthand panel) by *B. bacteriovorus* HD100 wild type (straight line fill), ΔBd0816 (diagonal line fill) and ΔBd3459 (solid fill) strains infecting *E. coli* S17-1 (pBd3460). Mean attachment time was measured from initial *Bdellovibrio* contact with the outside of prey cell to the start of traversal through the prey cell wall. Mean invasion time was measured from the start of traversal through the prey cell wall to not being visible outside the prey cell, that is, being completely within the prey cell. At least two independent experiments were carried out (*n*>50) with error bars showing 95% confidence intervals and statistical analyses shown (**P<0.01 ***P<0.001 in Student's *t*-test).

That Bd3460 does antagonize Bd3459 activity in an intracellular niche was shown by the observation that expression of Bd3460 in *E. coli* prey significantly reduced the rounding of prey when attacked by wild-type *Bdellovibrio* (Fig. 2a). This effect was also seen for mutant *Bdellovibrio* with single deletions of either *bd0816* or *bd3459*, providing evidence that activity of both DD-endopeptidases is antagonized by Bd3460.

High-level expression of Bd3459 from a vector with a tightly controlled promoter in *E. coli* cells has previously been shown to induce bacterial lysis^6^, with cells rounding up, swelling and finally bursting. Simultaneous co-expression of Bd3460 resulted in significant protection from the lytic effect of Bd3459 induction, with a larger proportion of *E. coli* cells remaining rod-shaped and growing by binary fission. That a proportion of the cells were still deformed and lysed shows that there was an imbalance of the two interacting species which led to active unbound Bd3459 protein damaging the cells (Supplementary Fig. 4).

### Predator Δ*bd3460* mutants self-round upon prey recognition

**Figure 3:**
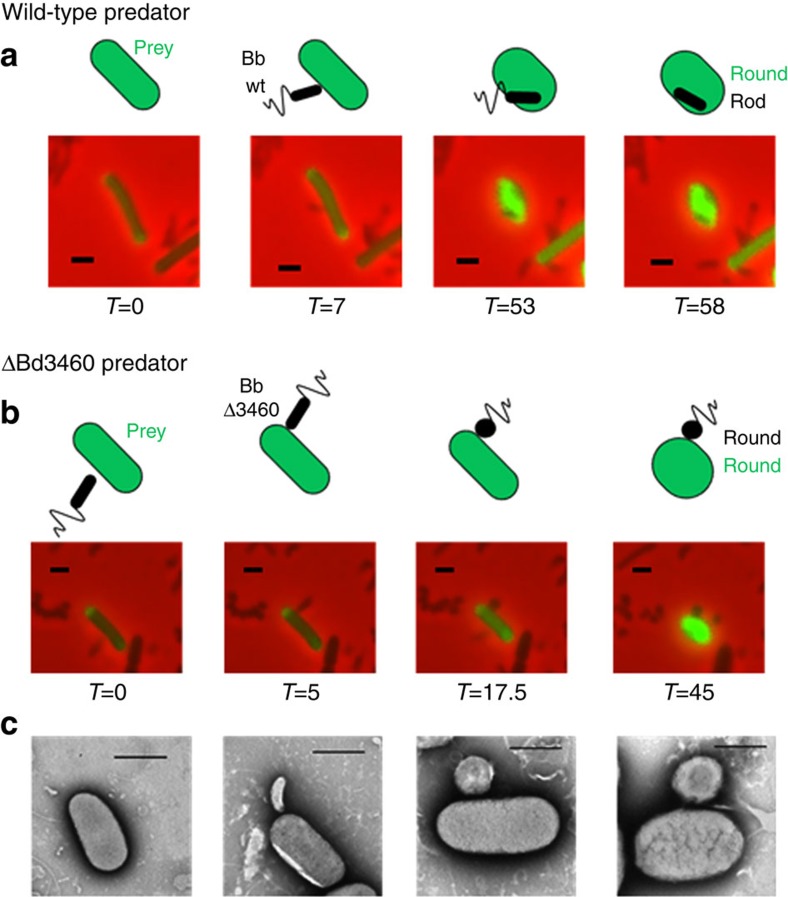
ΔBd3460 *Bdellovibrio* self-round upon initiating prey cell entry. Epifluorescence phase contrast microscopy of *Bdellovibrio* (small, phase dark, comma-shaped cells) preying upon *E. coli* prey cells which have periplasms constitutively fluorescently labelled by a pMal::mCherry fusion. A cartoon representation is presented above each. (**a**) Control using host independent strain HID22 which is wild-type for Bd3460 (Bb wt) and shows typical attachment to and entry into the prey cell which is rounded up in the process. (**b**) ΔBd3460 host independent strain (Bb Δ3460) attaches to the prey cell in a manner similar to the wild-type control, but then rounds up itself, preventing entry into the prey cell. (**c**) Representative electron micrographs showing the different stages of attachment, *Bdellovibrio* rounding, and prey rounding. Scale bars, 1 μm ; time is indicated in minutes.

Attempts to delete *bd3460* in predatory *Bdellovibrio* cells^14^, grown on prey, yielded only wild-type revertants or merodiploid strains. However *bd3460* deletions were readily obtained in HI (host/prey—independent) *Bdellovibrio* cells grown on artificial media without prey present. As *Bdellovibrio* does not prey on itself, there is no induction, under HI conditions, of *Bd3459/Bd0816*, thus the absence of Bd3460 was not detrimental in these circumstances. The HI strain with the *bd3460* deletion was then offered prey (HI *Bdellovibrio* do retain predatory abilities^14^). For wild-type *Bdellovibrio* this leads to prey binding, recognition and invasion (Fig. 3a). However, for the Bd3460 mutant there was a period of prey-binding and then after 41.5±26.5 min of attachment the *Bdellovibrio* cell suddenly (within 3.3±1.4 min) rounded up (example in Fig. 3b). Thus the Bd3459/Bd0816 DD-endopeptidase enzymes acted upon the self-cell wall of the *Bdellovibrio Δbd3460* mutant. These enzymes were still secreted from the *Δbd3460* mutant into the prey as evidenced by prey rounding (Fig. 3b). In addition, other damage was observed, such as leakage of the prey cell contents at the point of *Bdellovibrio* contact (Supplementary Fig. 5). This suggests that the predator was still breaching the prey outer membrane, but was unable to enter due to its own rounded deformation. The expression of either Bd0816 or Bd3459 from the *Bdellovibrio* was sufficient to cause predator self-rounding in the absence of Bd3460. Double mutants of Bd3460/Bd3459 and Bd3460/Bd0816 could only be isolated as host-independent isolates and also rounded up upon contact with prey cells (Supplementary Fig. 6). Triple mutants of *bd3460*/*bd3459*/*bd0816* were readily obtained and were capable of prey entry similar to wild-type in lab conditions (Supplementary Fig. 6). These observations fit with electron micrographs of prey entry, wherein wild-type *Bdellovibrio* is seen to deform and ‘squeeze' through an entry pore thinner than the predator cell width^15^,^16^.

### Structure of the Bd3460 self-protection ankyrin repeat protein

**Figure 4:**
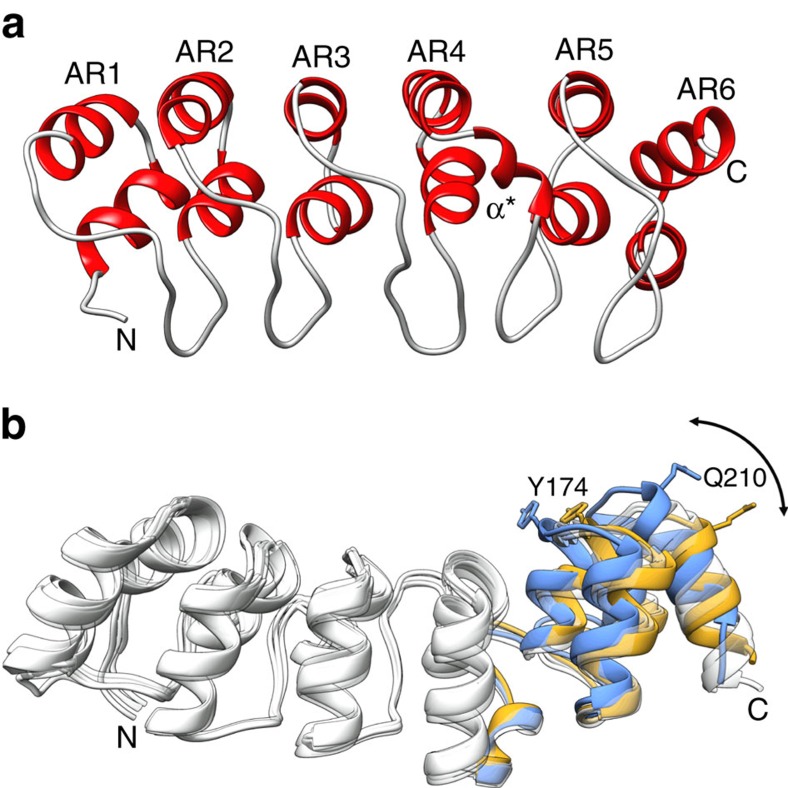
Structure of the endopeptidase self-protection protein Bd3460. (**a**) Sequential ankyrin repeats (AR) form the core of Bd3460, with a crossover helix (α*) between AR4/5; the AR4:AR5 packing differs from the other repeats, leading to a ‘4+2' arrangement. (**b**) View ∼90° from that in **a**, demonstrating relative flexation between the extended (orange, unbound chain A) and endopeptidase target-complexed (blue) forms of Bd3460, residues Y174 and Q210 represented in stick form to guide interpretation (AR1-4 conformation common to all forms, coloured white; chains B–E of unbound form represent states of intermediate conformation and are rendered transparent for clarity).

The structure of the exported, periplasmic region of Bd3460 (amino acids 26–220, hereafter referred to simply as Bd3460) was determined from X-ray diffraction data extending to 1.85 Å resolution (data collection and refinement statistics are provided in Supplementary Table 1). The structure is comprised of six sequential ankyrin repeats (AR1–AR6), which stack together to form the conventional ‘cupped hand' fold representative of ARPs, with the short β-strand ‘fingers' projecting out from the concave ‘palm' (Fig. 4a). The repeat regions largely conform to the 33 amino acid length of regular ARP motifs, with the longest loop present between AR3 and AR4 (Fig. 4a, sequence representation in Supplementary Fig. 7). The region surrounding the AR3:AR4 loop at the ‘centre' of Bd3460 displays three major deviations from the ankyrin structural consensus. Firstly amino acids V126 to G131 form an extended loop that differs from the usual tight turn present between the fingers of the other repeats of Bd3460/standard ARPs. Secondly, the crossover region at the end of AR4 (K154 to N158) is α-helical in nature much like that observed in the ARP IκBα (ref. 17). Thirdly, the repeats of Bd3460 can be grouped such that AR1:4 and AR5:6 stack with a regular angular periodicity, but AR5 is twisted with respect to AR4. This 4+2 arrangement of repeats results in a partial cleft between these two subdomains, lined by D132, M136, A139, Q140, A167, A170 and V171. The cumulative effect of the three deviations from consensus structure is that the central AR3:AR4 loop projects further from the core of Bd3460 than the other loops, and at a relatively more acute angle. Bd3460 displays conformational sampling such that chains A to E adopt different relative flexation between AR4 and AR5 (Fig. 4b).

### Architecture of Bd3460 in complex with multiple targets

**Figure 5:**
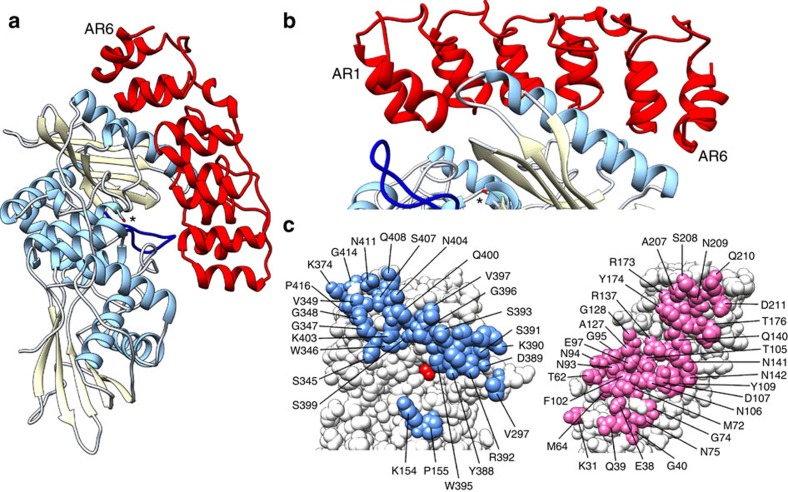
Details of the Bd3459 endopeptidase:Bd3460 auto-immunity protein complex. (**a**) An extensive interaction places all six ankyrin repeats of Bd3460 (ribbon form, red) over the upper, transpeptidase lobe of Bd3459 (ribbon form, helices sky blue, strands yellow; active site serine in stick form and denoted by asterisk; active site extended loop, dark blue). A small pocket of solvent exists at the protein interface. (**b**) Orthogonal view from a, demonstrating that AR1:6 of Bd3460 effectively wrap around the final helix of the Bd3459 transpeptidase domain, interacting via the helix-turn-helix section of the repeats. (**c**) Interacting partners have been rotated like an open book from orientation in a, Bd3459 90° to left, Bd3460 90° to right. The interaction face of Bd3459 (blue) is comprised of two continuous regions that abut, but do not comprise the active site cleft (catalytic serine coloured red). In contrast, the interacting face of Bd3460 (pink) is formed from a single face of the protein.

We next utilized a modified version of our original Bd3459 construct (starting post signal peptide with K38 mutated to become the new N-terminal methionine) that represents a more ‘native' signal peptide-processed form of the DD-endopeptidase^6^. This version (hereafter simply referred to as Bd3459), was co-expressed with the exported domain of Bd3460 and the structure of this complex determined to 1.36 Å resolution (Fig. 5a). The Bd3459:Bd3460 interaction reveals that AR1-3 of the self-protection protein are located over the endopeptidase active site cleft, whereas AR4-6 contact the final α-helix of the transpeptidase domain (α9, Fig. 5b). This orientation of binding situates the ankyrin ‘cupped hand' loops toward the rear face of the enzyme, such that the active site is blocked by the helix-turn-helix section of the AR repeats—this inhibition mode is ∼180° to that commonly observed for AR-mediated protein interactions, but has precedent in some ARP complexes e.g., p16Ink4a:Cdk6 (ref. 18).

The Bd3459:Bd3460 interface buries 2372 Å^2^ of surface area, and is largely polar in nature; 13 hydrogen-bonds, 1 salt bridge, 125 non-bonded contacts; indicating complexation largely via shape complementarity. Upon comparison with our structures of the uncomplexed proteins, Bd3459 does not alter in conformation upon binding, whereas Bd3460 undergoes further flexation around the region between AR4-AR5 (Fig. 4b). This observation explains the 4+2 arrangement of the repeats, such that the gap between AR4 and AR5 allows Bd3460 to undergo an induced fit and contact a patch of Bd3459 around the 346-351 loop and C-terminal end of helix α9. This interaction would not be possible if Bd3460 retained the extended conformation of the uncomplexed state. To the best of our knowledge, this significant conformational rearrangement following ARP target complexation is unique.

**Figure 6:**
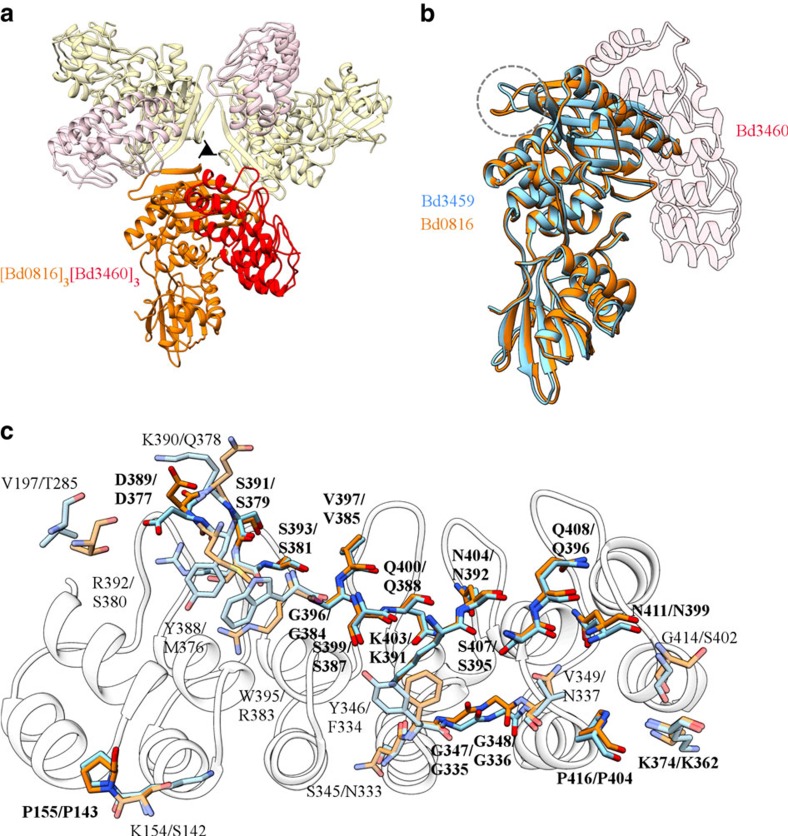
Complexation of the Bd3460 self-protection protein with the second endopeptidase partner Bd0816. (**a**) Heterohexameric Bd0816_3_:Bd3460_3_ complex, with a single pair (Bd0816, orange; Bd3460, red) in bold and remainder of hexamer transparent (Bd0816, yellow; Bd3460, pink). (**b**) Superimposition (using Bd3460, transparent) of the related Bd3459 and Bd0816 endopeptidases (blue and orange, respectively). The largest structural difference between the two targets forms part of the Bd0816 trimer interface (circled)—generally the folds show high equivalence. (**c**) Bd0816/Bd3459: Bd3460 interaction face comparison; Bd3459 (blue) and Bd0816 (orange) display a common, conserved interface (labels in bold type, standard stick form; located largely at the final transpeptidase domain α helix highlighted in Fig. 5b) at the core of the interaction, surrounded by a less conserved ‘halo' of variant interacting residues (labels in normal type, transparent stick form).

The use by *B. bacteriovorus* (and related periplasmic predators) of multiple DD-endopeptidases to decrosslink prey wall, and our observation of specific neutralization of Bd3459 by Bd3460 raised the question as to whether a similar mechanism is used to protect against Bd0816. Bd0816 expression was toxic but was circumvented by mutating the active site Serine residue to Alanine (S58A). The resulting 2.48 Å structure of the Bd0816:Bd3460 complex reveals a conserved mode of binding between the ARP self-protection protein and both predatory DD-endopeptidase targets (Fig. 6a,b).

The Bd0816:Bd3460 complex could be superimposed onto the Bd3459:Bd3460 co-ordinates with an RMSD for equivalent atoms of 0.76–0.94 Å (using different heterodimers of Bd0816:Bd3460 from the asymmetric unit), the conformation of Bd3460 being in agreement with the induced-fit observation outlined above. Bd0816 is largely structurally equivalent to Bd3459, with a few small differences in surface-exposed loops (Fig. 6b; loops involved in Bd0816 oligomerization). It is striking that we observe a trimeric form of Bd0816 (burying 1,041 A^2^ of surface area per monomer, on the borderline for statistically significant oligomers^19^). *In-vitro* characterization of the Bd0816:Bd3460 complex suggests the 1:1 heterodimer likely represents the dominant form in solution (Supplementary Fig. 8). Comparison of the residues involved in both complexes illustrates that the endopeptidase:self-protection protein interface is conserved (Fig. 6c), and is likely to be representative of protection mechanisms in related predators. The *B. bacteriovorus* housekeeping self endopeptidase Bd3244 (required for growth in walled bacteria) has notable sequence differences to the predation endopeptidases^6^, several of these map onto the Bd3460 interface unique to the predatory enzymes, indicating that self-wall maintenance would not be compromised by off-target complexation by Bd3460. The action of Bd3460 to neutralize two differing targets suggests intricate co-evolution, analysis of which will be revealed by large-scale sequencing of further predatory genomes.

The DD-endopeptidase substrate peptidoglycan is a large (and branched) molecule, and our structures indicate that the binding interface of the endopeptidase:Bd3460 complexes blocks substrate turnover without the need for extensive, active site-centered contacts (for example, like those observed in the β-lactamase-inhibitory protein:TEM β-lactamase complex^20^). The availability of the active site catalytic serine of the endopeptidase in the Bd3460-bound form (Fig. 5c) agrees well with the finding that acylation of Bd3459 with penicillin G had only a minor effect on complex formation. Indeed, we were able to demonstrate this further by acylating pre-grown Bd3459:Bd3460 complex crystals with penicillin G (Supplementary Fig. 9), hence Bd3459 retains activity and acylation propensity in complex and endopeptidase function is blocked via steric occlusion with Bd3460.

### Model for self-protection during prey invasion by predators

The *in vivo* and *in vitro* evidence suggest a model where Bd3460 is exported to the periplasm and persists there, acting to inhibit Bd3459 and Bd0816 function after folding in the periplasm and before reaching the cell wall target in prey. The protein complexes show that the interaction face involves the signal peptide cleavage site for Bd3459/Bd0816, hence binding may potentially be strongest to the mature form of these enzymes and thus not interfere with secretion and/or processing by signal peptidase, with Bd3460 forming protective complexes only in the periplasm. The simplest scenario for self-protection, coupled to effective rounding of prey, would be to retain Bd3460 and differentially secrete Bd3459/Bd0816. The induced fit of Bd3460 upon binding the DD-endopeptidases could potentially be exploited to lessen any interaction and aid Bd3459/Bd0816 ‘stripping' during final export into prey. Periplasmically retained Bd3460 (visualized as an abundant Bd3460:mCherry fluorescent signal) would be able to inhibit Bd0816, compensating for its expression from its genetic locus outside of the *bd3459*/*bd3460* operon.

Heterologous expression of Bd3460 in prey did not abolish predator entry (as for the double endopeptidase mutant *Bdellovibrio*) although deletion of Bd3460 in *Bdellovibrio* produced a cell that puffed up and did not enter prey. Furthermore it is clear that prey naturally acquiring the gene to express Bd3460 in their periplasm would not be immune from *Bdellovibrio* entry, consistent with the observations that *Bdellovibrio* have a wide prey range and that natural resistance is not seemingly easily acquired^4^. The ‘puffing up' of the ΔBd3460 *Bdellovibrio* after a period of binding to and recognizing the prey cell may represent a useful tool to discern prey recognition.

The acquisition/source of the *bd3460* gene during the evolution of invasive predatory *Bdellovibrio* is as yet unknown. The ankyrin repeat residue conservation complicates homology searches, but the highest-ranked BLAST hit for Bd3460 from a non-predatory bacterium is an ARP from the spirochete *Leptospira kirschneri* (UniProt accession code K6IH47; in an operon with a protease). Ancient lateral gene transfer to the *Bdellovibrio* genome of genes from spirochaete genomes has been noted by Gophna *et al*.^21^ and is predicted to represent key stages in the evolution of predation. The acquisition and diversification of an ancient ARP may have increased selection for the gene duplication and diversification of a housekeeping DD-endopeptidase (like the modern Bd3244 for self-wall modification and growth) and its recombination at the ARP gene locus. Ultimate co-expression of the two may have allowed the predatory DD-endopeptidase gene(s) to evolve from the housekeeping form without causing damage to the predator cell wall. The prevalence of ARPs as effectors in intracellular parasites and symbionts such as *Coxiella*, *Wolbachia* and *Legionella* is also of note^10^,^11^, and a recent report detected homology between the mevalonate-metabolizing proteins of *Bdellovibrio* and *Legionella pneumophila*, suggesting that gene transfer between the two is possible^22^. Other ARPs can be identified in the *B. bacteriovorus* HD100 genome, one of which (Bd1180) is in an operon with the peptidoglycan LD-transpeptidase Bd1181 (ref. 23), hence our identification of ankyrin repeat protein Bd3460 as a key player in self-protection may lead us to identify important enzymes in the predatory process by locating putative immunity protein:effector pairs and we predict that Bd1181 and Bd1180 will act in a similar paired way to manipulate prey while protecting self.

In summary, we conclude that we have identified and characterized the first ever self-protection protein encoded by predatory bacteria—ankyrin repeat protein Bd3460 from *Bdellovibrio* inhibits the prey wall decrosslinking enzymes Bd3459/Bd0816 and in doing so protects the predator from the shape transition that this catalyses in prey. The Bd3459/Bd0816:Bd3460 relationship is integral to *Bdellovibrio* predation, regulating prey entry and self-protection (Bd3460) and also niche formation and population fitness (Bd3459/Bd0816). We therefore regard the Bd3459/Bd0816:Bd3460 interaction as a key predatory adaptation and a significant step in understanding the hierarchical biochemical timeline of staged prey recognition and invasion and the evolution of an intracellular lifestyle.

## Methods

### RNA isolation from predatory cycle and RT-PCR analysis

Synchronous predatory infections of *B. bacteriovorus* HD100 by predation with MOI>2 in 100 ml 2 mMCaCl_2_/25mMHEPES buffer pH 7.6 on *E. coli* S17-1 as well as S17-1 alone were set up as previously described^24^. Samples were taken throughout the timecourse and total RNA isolated from them. RNA was isolated from the samples using a Promega SV total RNA isolation kit with the RNA quality being verified by an Agilent Bioanalyser using the RNA Nano kit. RT-PCR was performed with the Qiagen One-step RT-PCR kit with the following reaction conditions: One cycle 50 °C for 30 min, 95 °C for 15 min, then 25 cycles of 94 °C for 1 min, 48 °C for 1 min, 72 °C for 2 min and finally a 10 min extension at 72 °C after the 30 cycles, and finally a 4 °C hold. All experiments were carried out with at least two biological repeats. Primers used to anneal to *bd3460* were 5′-TTTCCTCGCGGGCCTTCTGC-3′ and 5′-GGCCAGATCACCTTGTTCCGCC-3′. Primers used to anneal to *bd3459* were 5′-ACAAGTCCCGCTCTGACTGGG-3′ and 5′-GTACTTGATTGCTTTTGGTCCGCCG-3′.

### Fluorescent tagging of Bd3460

The *bd3460* gene was cloned into the pK18*mobsacB* mobilizable vector in such a way as to fuse the gene at the C-terminus with the mCherry gene, using the NEB Gibson assembly kit. The primers used to amplify *bd3460* were: 5′-CGACGGCCAGTGCCAATGAAAAAATCCTATCTGCTG-3′ and 5′-CTCACCATTTTCTTTTTGGAGAGAGCTTTTG-3′ and to amplify *mcherry: 5*′-CAAAAAGAAAATGGTGAGCAAGGGCGAG-3′ and 5′-CTATGACCATGATTACGTTACTTGTACAGCTCGTCCATG-3′. This construct was introduced to *Bdellovibrio* via conjugation from *E. coli* S17-1 donor strain, mating overnight at 29°C with *B. bacteriovorus* HD100 on a nitrocellulose filter on a PY (10 g l^−1^ peptone, 3 g l^−1^ yeast extract) agar plate, before selection for exconjugants by 50 μg ml^−1^ kanamycin sulphate in YPSC double layer agar plates (1 g l^−1^ peptone, 1 g l^−1^ yeast extract, 0.5 g l^−1^ anhydrous sodium acetate, 0.25 g l^−1^ M_g_SO_4_.7H_2_O, pH 6.8), as described previously^25^,^26^.

### Heterologous expression of Bd3460 and roundness measurement

Phase contrast time-lapse microscopy was carried out on predation by *Bdellovibrio* of *E. coli* S17-1 harbouring the pET26b expression vector with the *bd3460* gene under the control of an IPTG inducible promoter. The prey *E. coli* were grown in YT broth (8 g l^−1^ bacto-tryptone, 5 g l^−1^ yeast extract, 5 g l^−1^ NaCl, pH 7.5) for 16 h with shaking at 200 r.p.m. with kanamycin sulphate selection at 50 μg ml^−1^ either with or without 200 μg ml^−1^ IPTG induction before being washed and concentrated five times in Ca/HEPES buffer by centrifugation for 2 min at 17,000*g*. *Bdellovibrio* cultures were grown for 16 h as a prey lysate in 50 ml Ca/HEPES buffer on 3 ml *E. coli* S17-1 prey as described previously^22^,^24^ before being concentrated 50 times in Ca/HEPES buffer by centrifugation for 2 min at 17,000*g*. The concentrated preparations of predator and prey were mixed and immediately added to a microscope slide with a layer of 0.3% agarose in Ca/HEPES buffer to immobilise the prey cells. Immobilized cells were visualized using a Nikon Eclipse E600 microscope using a × 100 objective lens (numerical aperture (NA), 1.25) and an exposure time of 0.1 s. Images were acquired using a Hamamatsu Orca ER camera and the Simple PCI software (version 6.2 from Digital Pixel). An H101A *xy* motorized stage (Prior Scientific) allowed precise revisiting of different locations on the slide (minimum step size, 0.01 μm), and a frictional *z*-axis controller (minimum step size, 2 nm) in conjunction with the Simple PCI software allowed fine autofocusing on immobilized developing bdelloplasts. Images were enhanced using either (or both) the ‘sharpen' and ‘smooth' tools in the Image J software to provide additional clarity. To investigate the differing bdelloplast shapes quantitatively, traces of the bdelloplasts in 90 min images were made and measures of ellipticity were taken using the Image J software to find an average ‘roundness' coefficient for each invading *Bdellovibrio* strain and prey combination.

### Co-expression of Bd3459 and Bd3460 in *E. coli*

The *bd3460* gene was introduced into the pET26b vector by recombineering, using the primers 5′-CCTCGCTGCCCAGCCGGCGATGGCCTCAGGGAAGTCCAGCAAGGCCTTG-3′ and 5′-CTCAGTGGTGGTGGTGGTGGTGCTCGAGTTATTTCTTTTTGGAGAGAGCTTTTGC-3′. An apramycin resistance cassette was then introduced in place of the kanamycin resistance cassette. The Bd3459 construct in the pBADHisA vector, with Bd3459 under control of a promoter inducible by arabinose, has been described previously^6^, and was modified to introduce an apramycin resistance cassette in place of the ampicillin or kanamycin resistance cassette. The two vectors could then be maintained in *E. coli* with apramycin and kanamycin selection. *E. coli* Top10 (Bd3459pBADapra+Bd3460pET26bkan) were backdiluted to OD_600_ of 1.0, pre-incubated 1 h with selection (with or without IPTG), then 10ul was put on pads consisting of 1% agarose YT+0.2% arabinose on slides for microscopy with images acquired every 150 s with the microscopy setup as above. Images at every hour were then analysed by manually scoring cells as intact or damaged and numbers were expressed as a percentage. As a result of cell growth, it was not possible to accurately score images after 4 h as large damaged cells merged together. A minimum of two biological repeats and *N*=220–1,062 for each timepoint, with more cells counted at the later timepoints. *T*-test gave values of *P*<0.001 for *t*=2, 3 and 4 h.

### Cloning of Bd3460 and co-expressed endopeptidases

Overexpression constructs were generated by a restriction-free cloning strategy^27^. In brief, nucleotide primer pairs complementary to both the target gene (at the 3′ end of each primer) and destination vector (at the 5′ end of each primer) were used to generate PCR products which were subsequently inserted into plasmids by a second PCR reaction.

Primer pairs 5′-GTTTAACTTTAAGAAGGAGATATACATATGTCAGGGAAGTCCAGCAAGGCCTTG-3′ and 5′-GTGGTGGTGGTGGTGGTGCTCGAGTTTCTTTTTGGAGAGAGCTTTTGC-3′ were used to amplify the region encoding the predicted secreted form of Bd3460 (starting at Ser26 with mutation of Ala25 to become the new N-terminal methionine, and placing a non-cleavable LEH_8_ peptide tag on the C-terminal end of the protein) for cloning into a modified version of the expression plasmid pET41 (Novagen, altered to remove glutathione S-transferase (GST)).

A new variant of the predicted secreted form of Bd3459 was also cloned into modified pET41 using primers 5′-TTTAACTTTAAGAAGGAGATATACATATGAAAGTTTACTTGAATTCCATGTGCC-3′ and 5′-TTAGTGGTGGTGGTGGTGGTGGTGGTGCTCG AGTTTCTTCTCTGTCGTGATAGTGTTC-3′, yielding a construct similar to that described previously but starting with codon K38M as opposed to A37M^6^. Bd0816 was cloned into modified pET41 using primers 5′-TTTAACTTTAAGAAGGAGATATACATATGGTTTATGTCAATTCCGTCTG-3′ and 5′-TTAGTGGTGGTGGTGGTGGTGGTGGTGCTCGAGCTTCTTGGAAAGATTCACAAC-3′, starting at K26M.

For co-expression of proteins, Bd3460 was cloned into pCDF-Duet1 (Novagen) using primers 5′-GTTAAGTATAAGAAGGAGATATACATATGTCAGGGAAGTCCAGCAAGGCC-3′ and 5′-GGTGGCAGCAGCCTAGGTTAATTATTTCTTTTTGGAGAGAGCTTTTGC-3′. The resultant plasmid, which contained Bd3460 sequences as described above (but not fused to a purification tag) was used as the destination vector for sequential cloning of Bd3459 (5′-GTTTAACTTTAAGAAGGAGATATACCAT GGTTTACTTGAATTCCATGTGCCATATGG-3′ and 5′-CGATTACTTTCTGTTCGACTTAAGCATTAGTGGTGGTGGTGGTGGTGGTGGTGCTC-3′) or Bd0816 (primers 5′ GTTTAACTTTAAGAAGGAGATATACCAT GGTTTATGTCAATTCCGTCTG-3′ and 5′-CGATTACTTTCTGTTCGACTTAAGCATTAGTGGTGGTGGTGGTGGTGGTGGTGCTC-3′). Bd3459 and Bd0816 fragments for this second cloning stage were amplified from the corresponding pET41 derived plasmids to provide C-terminally H_8_ tagged endopeptidases.

The Bd3459 S70A and Bd0816 S58A mutants were generated via standard Quikchange protocol (Stratagene). All constructs were confirmed by sequencing, and introduced into the *E. coli* expression strain T7 express (New England BioLabs).

### Protein expression and purification

For purification of Bd3460, cells were grown at 37 °C until reaching an OD_600_ of ∼0.6, then gene expression induced with 1 mM IPTG for 20 h at 20 °C. Harvested cells (∼12 g from 1 l cell culture in TB medium) were resuspended by tumbling in 45 ml resuspension buffer (20 mM Hepes pH 7.2, 0.25M NaCl, 5% w/v glycerol, 20 mM imidazole and 10 mM sodium cholate) and lysed using sonication. Unbroken cells were pelleted by centrifugation at 6,000*g* for 20 min, the supernatant clarified by a second centrifugation at 200,000*g* for 1 h and the final supernatent applied to a 1 ml Hi-Trap His column, pre-equilibrated in modified buffer A (lacking sodium cholate). Fractions were eluted in a stepwise manner, using buffer A containing 40 and 300 mM imidazole. Approximately pure fractions of Bd3460 were dialysed overnight in buffer B (10 mM Hepes pH 7.2, 0.25 M NaCl) and concentrated to a protein concentration of ∼20 mg ml^−1^. Bd3459 (original construct) and Bd3459 new variant (K38M start, S70A) were both expressed and purified as reported previously for Bd3459 (ref. 6).

Similar strategies were employed for the overexpression and purification of the Bd3459/Bd3460 and Bd0816 S58A/Bd3460 complexes. Buffer C (20 mM imidazole pH 8.0, 0.4 M NaCl, 0.05% w/v Tween20) was used for resuspension of cells and in the place of buffer A for the purification of complexes. Purified complexes were dialysed overnight into buffer D (20 mM Bis-Tris pH 6.5, 0.2 M NaCl) and were concentrated to ∼25 and ∼30 mg ml^−1^ for the Bd3459/Bd3460 and Bd0816 S58A/Bd3460 complexes, respectively.

Analytical gel filtration experiments were performed on a HiLoad 26/60 Superdex 200 column (GE Healthcare) using buffer D (20 mM Bis-Tris pH 6.5, 0.5 M NaCl).

### Crystallization and structure determination

Crystals were grown by the hanging drop method at 18 °C, using 1 μl of protein solution mixed with an equal volume of reservoir solution. Initial apo-Bd3460 crystallization conditions were identified in Midas screen II condition #27 (40% v/v glycerol ethoxylate^28^). Crystals of Bd3459 S70A were grown in JCSG-*plus* screen II condition #33 (0.1 M potassium thiocyanate; 30% w/v PEG 2000 MME). The Bd3459/Bd3460 and Bd0816 S58A/Bd3460 complexes crystallized in JCSG-*plus* screen II conditions #14 (0.1 M citrate, pH 5.0; 3.2 M ammonium sulphate) and #47 (0.1 M Hepes, pH 7.5; 0.2 M MgCl_2_; 25% w/v PEG 3350), respectively. Crystals of the Bd3459^M^/Bd3460 complex were incubated with reservoir solution supplemented with 2 mM penicillin G for one hour to yield an additional, acylated complex structure.

All crystals were directly flash cooled in liquid nitrogen and diffraction data were collected at the Diamond Light Source, Oxford. Data were processed using XDS^29^ and SCALA, and data file manipulations performed using the CCP4 suite of programs^30^. A heavy atom derivative was obtained by growing Bd3460 crystals directly in the presence of 300 mM Potassium Iodide (data collected on a home source, CuK_α_, Rigaku Micromax generator). Phasing of the derivative was accomplished using a combination of SHARP^31^ and PHENIX^32^ (FOM of 0.43, 20 I sites); the resultant phases were improved by applying manually derived non-crystallographic symmetry operators (five independent chains are present in the unit cell). After autobuilding in PHENIX, the remaining parts of the molecule were built manually using COOT^33^ and model refinement used PHENIX^32^ and the PDB-REDO server^34^. Complex structures were phased using molecular replacement with the Bd3460 and Bd3459^M^ isolated structures and the program PHASER^35^ and built/refined as outlined above. The final models are of excellent stereochemical quality (Table 1).

The closest non-synthetic structural neighbor of Bd3460 (as calculated by DALI^36^) is the uncharacterized ARP EF0377 from *Enterococcus faecalis* (PDB 3hra, RMSD 2.5 Å over 167AA alignment), although Bd3460 shares slightly higher structural homology to various DARPins (designed ankyrin repeat proteins, engineered for affinity purposes, for example, PDB codes 4hb5, 3nog).

All structural figures were generated using the program Chimera^37^.

### Enzyme activity measurements

Incubation of Bd3459 with isolated sacculi (± the presence of Bd3460) and subsequent HPLC analysis of cellosyl products (muropeptides) utilized an identical protocol to that documented in the original *Bdellovibrio* endopeptidase study^6^.

### Tryptophan fluorescence ligand binding measurements

Intrinsic tryptophan fluorescence ligand binding experiments were carried out using a Hitachi F-7000 fluorescence spectrophotometer. The excitation wavelength was set at 280 nm and the fluorescence emission (*F*_emission_) spectra was recorded between 300–400 nm. Purified Bd3459 was diluted with buffer B (Hepes switched to Tris) to a final concentration of 10 μM loaded into a quartz cuvette (final volume of 400 μl) equilibrated to a chamber temperature of 25 °C. Bd3460 was sequentially titrated against Bd3459 with *F*_emission_ recorded between each addition. On occasions, 0.75 mM penicillin G was pre-incubated with Bd3459 before being titrated with Bd3460. GraphPad Prism software was used to plot the change in fluorescence emission *(ΔF*_emission_) at λ_340nm_ versus [Bd3460] and data were fitted to a one site-specific binding isotherm *(ΔF*_emission_=*F*_max_ × *L*/(*K*_d_+*L*), where *F*_max_ indicates the maximum change in fluorescence emission, *K*_d_ is the binding constant and *L* is the concentration of ligand (Bd3460).

## Additional information

**Accession codes:** Crystallographic data have been deposited in the RCSB Protein Data Bank under accession codes 5CEA (Bd3460), 5CEB (Bd3459 K38M new construct), 5CEC (Bd3459:Bd3460 complex), 5CED (Bd3459:Bd3460 complex acylated with penicillin G) and 5CER (Bd0816:Bd3460 complex).

**How to cite this article:** Lambert, C. *et al*. Ankyrin-mediated self-protection during cell invasion by the bacterial predator *Bdellovibrio bacteriovorus*. *Nat. Commun.* 6:8884 doi: 10.1038/ncomms9884 (2015).

## Supplementary Material

Supplementary Information Supplementary Figures 1-9

## Figures and Tables

**Table 1:** Data collection and refinement statistics.

	**Bd3460 native**	**Bd3460 iodine derivative**	**Bd3459 K38M,S70A variant**	**Bd3459:Bd3460 complex**	**Bd0816:Bd3460 complex**	**Bd3459:Bd3460 PenG**
Accession code	5CEA	—	5CEB	5CEC	5CER	5CED
*Data collection*						
Space group	P2_1_2_1_2_1_	P2_1_2_1_2_1_	P1	P2_1_2_1_2_1_	P2_1_2_1_2	P2_1_2_1_2_1_
Cell dimensions						
*a*, *b*, *c* (Å)	57.43, 99.64, 173.70	57.13, 99.18, 173.33	55.93, 65.04, 73.82	51.61, 59.17, 192.27	212.32, 237.45, 90.90	51.54, 59.16, 192.04
*α*, *β*, *γ*(°)	90, 90, 90	90, 90, 90	63.89, 83.27, 83.20	90, 90, 90	90, 90, 90	90, 90, 90
Resolution (Å)	1.85 (1.95–1.85)*	2.4 (2.53–2.4)	1.93 (1.98–1.93)	1.36 (1.4–1.36)	2.48 (2.54–2.48)	2.02 (2.07–2.02)
*R*_sym_	7.0 (49.3)	6.3 (31.0)	3.6 (76.6)	3.2 (51.6)	15.6 (75.3)	6.7 (67.2)
*R*_pim_	6.0 (42.9)	1.6 (26.7)	2.5 (54.9)	2.0 (41.9)	4.6 (21.5)	3.1 (30.8)
CC 1/2†	0.99 (0.65)	0.99 (0.79)	0.99 (0.78)	0.99 (0.78)	0.99 (0.85)	0.99 (0.80)
*I*,/σ*I*	10.0 (2.5)	34.4 (2.7)	16.2 (1.6)	23.4 (2.4)	15.1 (3.8)	16.2 (2.8)
Completeness(%)	99.0 (100.0)	89.5 (47.8)	85.7 (79.2)	97.2 (77.8)	99.0 (99.5)	99.9 (99.9)
Redundancy	3.8 (3.7)	13.9 (2.0)	3.8 (3.7)	6.0 (3.7)	13.5 (14.0)	6.5 (6.7)
						
*Refinement*						
Resolution (Å)	1.85	—	1.93	1.36	2.48	2.02
*R*_work_/*R*_free_	17.8/21.0	—	19.8/23.2	14.3/16.9	21.7/24.9	17.3/20.7
r.m.s. deviations						
Bond lengths (Å)	0.017	—	0.016	0.011	0.014	0.014
Bond angles (°)	1.75	—	1.63	1.44	1.68	1.60

^*^Values in parentheses are for highest resolution shell.

^†^CC 1/2 is the correlation coefficient between two random half data sets.
