# Erratum: Ankyrin-mediated self-protection during cell invasion by the bacterial predator *Bdellovibrio bacteriovorus*

**DOI:** 10.1038/ncomms10483

**Published:** 2016-01-21

**Authors:** Carey Lambert, Ian T. Cadby, Rob Till, Nhat Khai Bui, Thomas R. Lerner, William S. Hughes, David J. Lee, Luke J. Alderwick, Waldemar Vollmer, R. Elizabeth Sockett, Andrew L. Lovering

Nature Communications **6**: Article number: 888410.1038/ncomms9884 (2015); Published: 12022015; Updated: 01212016.

The original HTML version of this Article contained an error in the spelling of the author R. Elizabeth Sockett, which was incorrectly given as Elizabeth R. Sockett. This has now been corrected in the HTML. The PDF version of the Article was correct from the time of publication.

