# Supplementary material for: Ankyrin-mediated self-protection during cell invasion by the bacterial predator Bdellovibrio bacteriovorus
Source: Nat Commun. 2015 Dec 2;6:8884. doi: 10.1038/ncomms9884 (PMC4686830; doi:10.1038/ncomms9884)
Supplement: Supplementary Information — Supplementary Figures 1-9 [file ncomms9884-s1.pdf]

Supplementary Material for Lambert *et al* "Ankyrin-Mediated Self-Protection During Cell Invasion by a Bacterial Predator"

Supplementary Figures

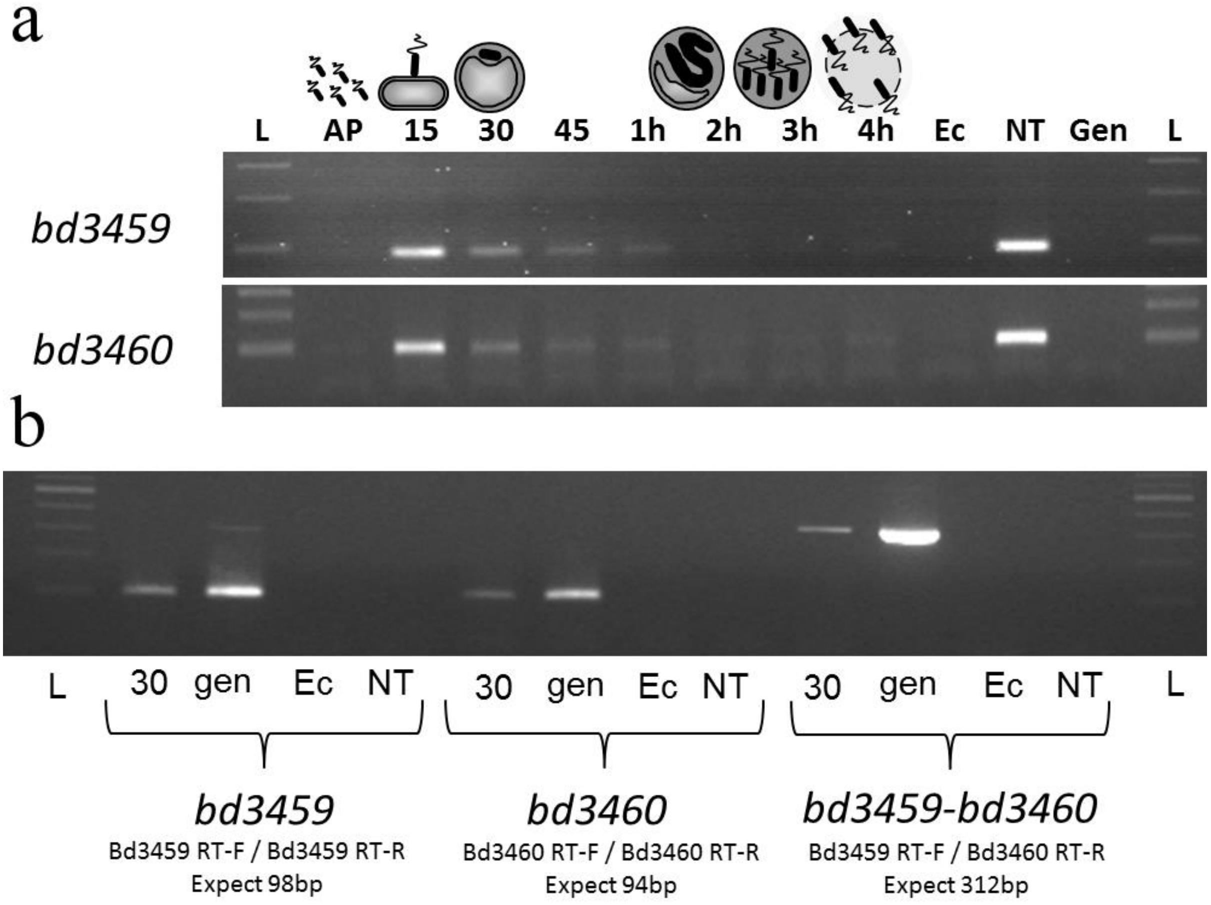

Supplementary Figure 1 | *bd3459* and *bd3460* are co-transcribed and comprise a true operon. a, Reverse Transcriptase PCR showing the expression of *dacB* endopeptidase gene *bd3459* and gene *bd3460* over the predatory cycle of *Bdellovibrio bacteriovorus*. RNA was isolated at time points throughout one round of synchronous *Bdellovibrio* infection of *E. coli* cells. A schematic of the different stages of infection is displayed above. Expression of both genes peaks at 15 to 30 minutes. AP = Attack Phase *Bdellovibrio*; 15, 30, 45 = minutes since start of infection (attachment to and rounding of *E. coli* host cell into a bdelloplast structure); 1h, 2h, 3h, 4h = hours since start of infection (host cell resources being degraded and used for

Bdellovibrio growth into a filament, followed by septation into multiple progeny and eventually lysis from host cell); Ec = *E. coli* S17-1 RNA (no *Bdellovibrio* control); gen = *B. bacteriovorus* HD100 genomic DNA (positive control); NT = No template control; L = NEB 100 bp DNA ladder. **b,** Reverse transcriptase PCR showing that *bd3459* is co-transcribed with *bd3460* in an operon. RT-PCRs using primers specific to a C-terminal region of *bd3459* (Bd3459 RT-F and Bd3459 RT-R, yielding a 98 bp product) and an N-terminal region of *bd3460* (Bd3460 RT-F and Bd3460 RT-R, yielding a 94 bp product) amplified an internal fragment of each gene from *B. bacteriovorus* HD100 RNA isolated after 30 minutes post-infection of *E. coli* prey (represented by “30”). Using the same RNA template but with Bd3459 RT-F and Bd3460 RT-R primers together produced the expected 312 bp product that would only be produced if there was a continuous mRNA transcript between the two genes confirming that Bd3459 and Bd3460 are in an operon together. gen = *B. bacteriovorus* HD100 genomic DNA, used as a positive control. Ec = *E. coli* S17-1 RNA (no *Bdellovibrio* control); NT = No template control; L = NEB 100 bp DNA ladder.

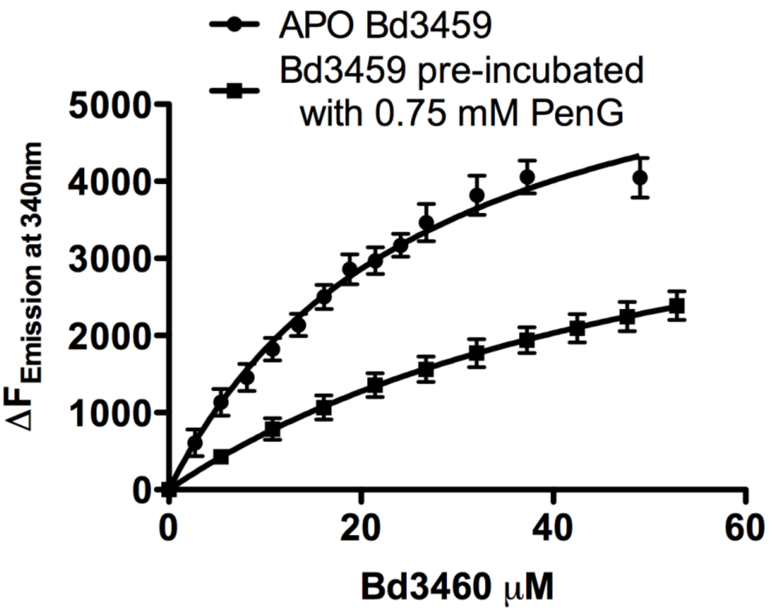

Supplementary Figure 2 | Fluorescence measurement of Bd3459:Bd3460 Interaction.

10 µM of Bd3459 (in the absence or presence of 0.75 mM Penicillin G) was titrated with Bd3460 and emission spectra recorded between 300-400 nm after excitation at 280 nm. Increasing concentrations of Bd3460 resulted in increased quenching of intrinsic tryptophan fluorescence of Bd3459 in its Apo (unbound) form. This effect was reduced approximately 2-fold upon pre-incubation of Bd3459 with Penicillin G.

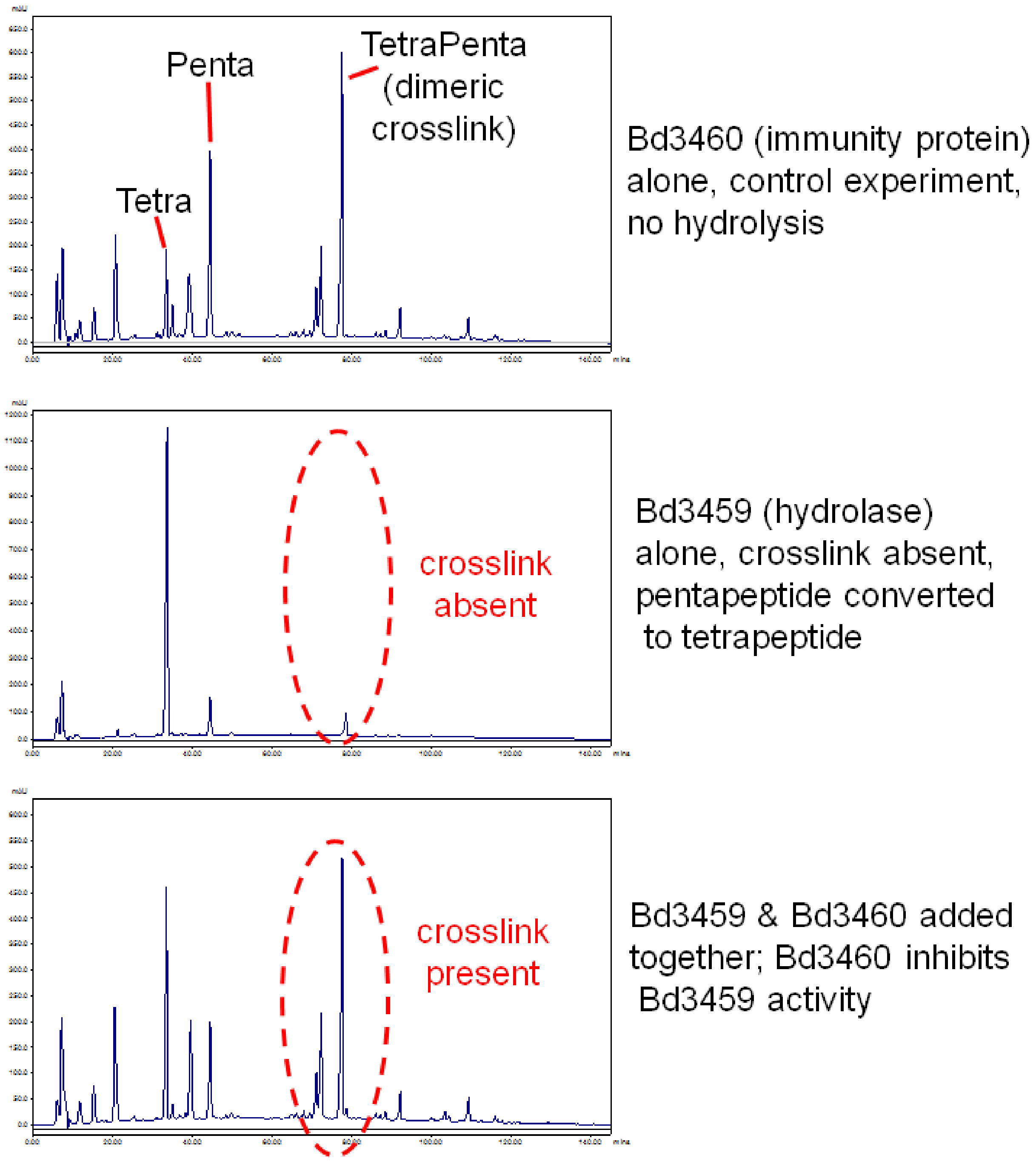

Supplementary Figure 3 | Peptidoglycan hydrolysis assay for Bd3460 auto-immunity function. HPLC traces of isolated peptidoglycan incubated with: (top, Bd3460 alone; middle, Bd3459 alone; bottom, Bd3459 and Bd3460 in tandem), followed by digestion with cellosyl and reduction of the resulting muropeptides by sodium borohydride. Comparison of the three traces indicates protective inhibitory effect of Bd3460 against Bd3459 endopeptidase activity. Muropeptides: Tetra, disaccharide tetrapeptide; penta, disaccharide pentapeptide; TetraPenta,

bis-disaccharide tetrapentapeptide (cross-linked muropeptide). There is some residual carboxypeptidase activity in the complex sample (penta to tetra conversion), which can be attributed to the smaller nature of this as a substrate *c.f.* endopeptidase/crosslinks, and the accessibility of S70 in complex (Figure 5a, Supplementary Figure 8).

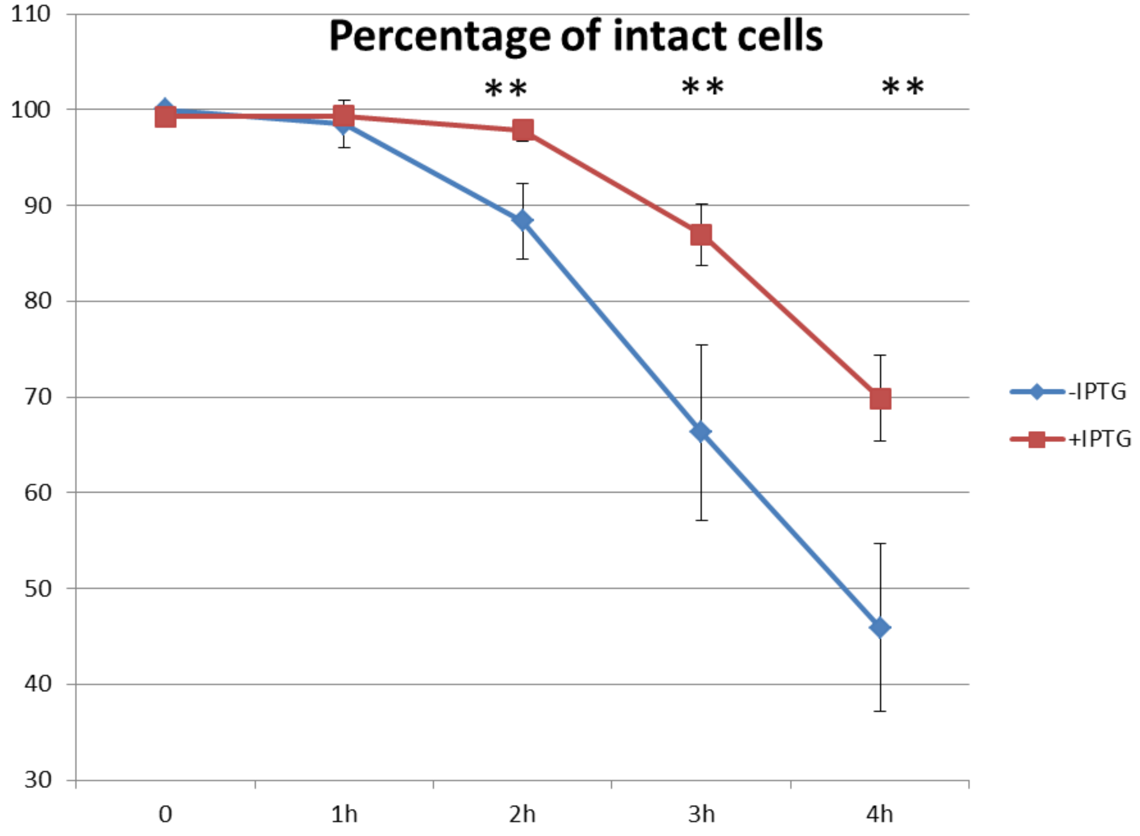

Supplementary Figure 4 | Heterologous Bd3460 protects *E. coli* from effects of Bd3459 expression. Graph showing percentage of intact *E. coli* cells heterologously expressing both Bd3459 and Bd3460 on separate plasmids. Bd3459 was induced by 0.2% arabinose in all cells. Bd3460 induction by IPTG (red squares) protected more of the cells from the damage caused by Bd3459 induction relative to those without IPTG induction (blue diamonds). Error bars are 95% confidence intervals. **- p<0.001 by Student's T-test.

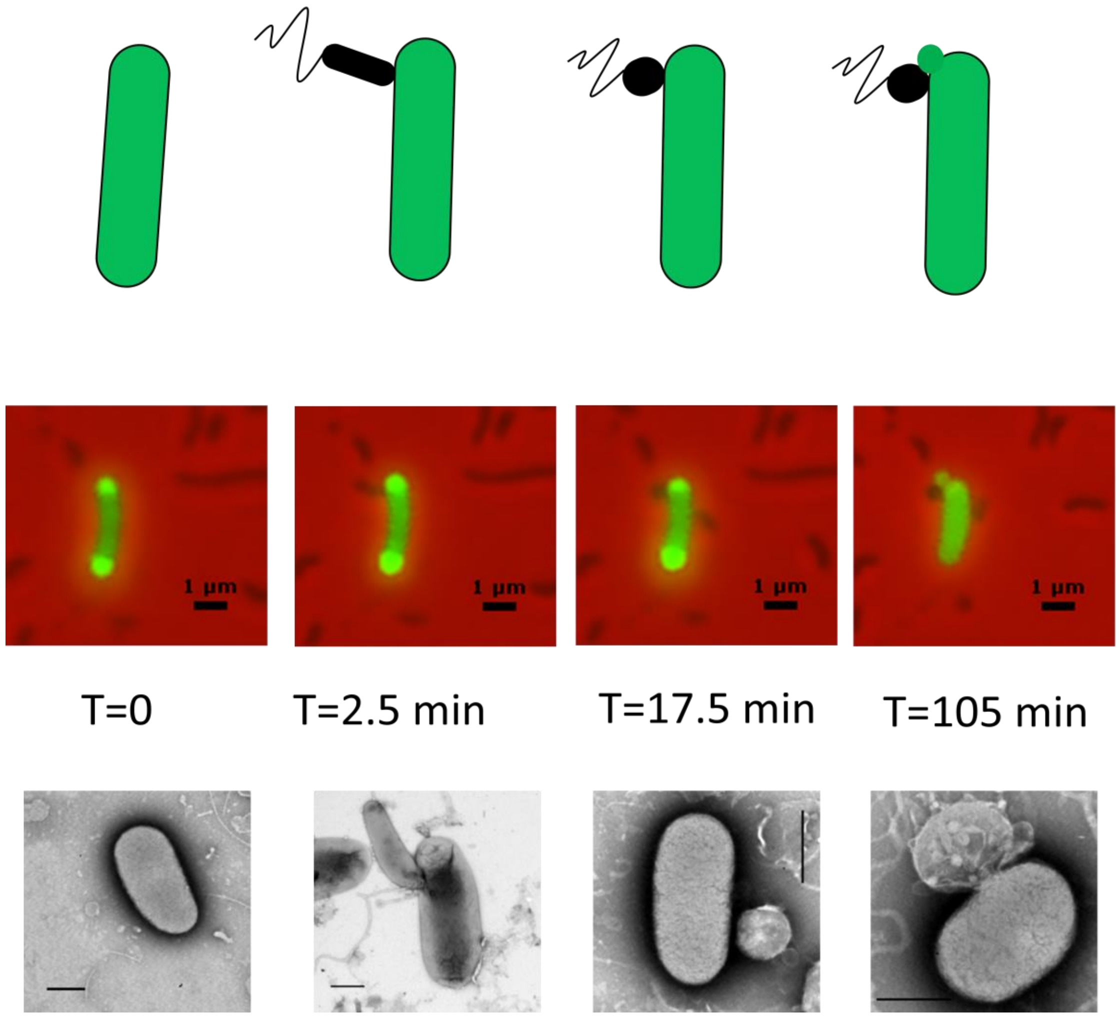

Supplementary Figure 5 | Visualization of prey cell damage. Epifluorescence phase contrast microscopy of *Bdellovibrio* (small, phase dark, comma-shaped cells) attaching to *E. coli* prey cells which have their periplasm constitutively fluorescently labeled by a pMal::mCherry fusion. A cartoon representation is presented above and representative electron micrographs are shown below. ΔBd3460 host independent strain attaches to the prey cell in a manner similar to the wild-type control, but then rounds up itself, preventing entry into the prey cell. Damage to the prey cell is seen as fluorescent material leaks out at the point of *Bdellovibrio* attachment (rightmost panel). Scale bars are 1μm and time is indicated in minutes.

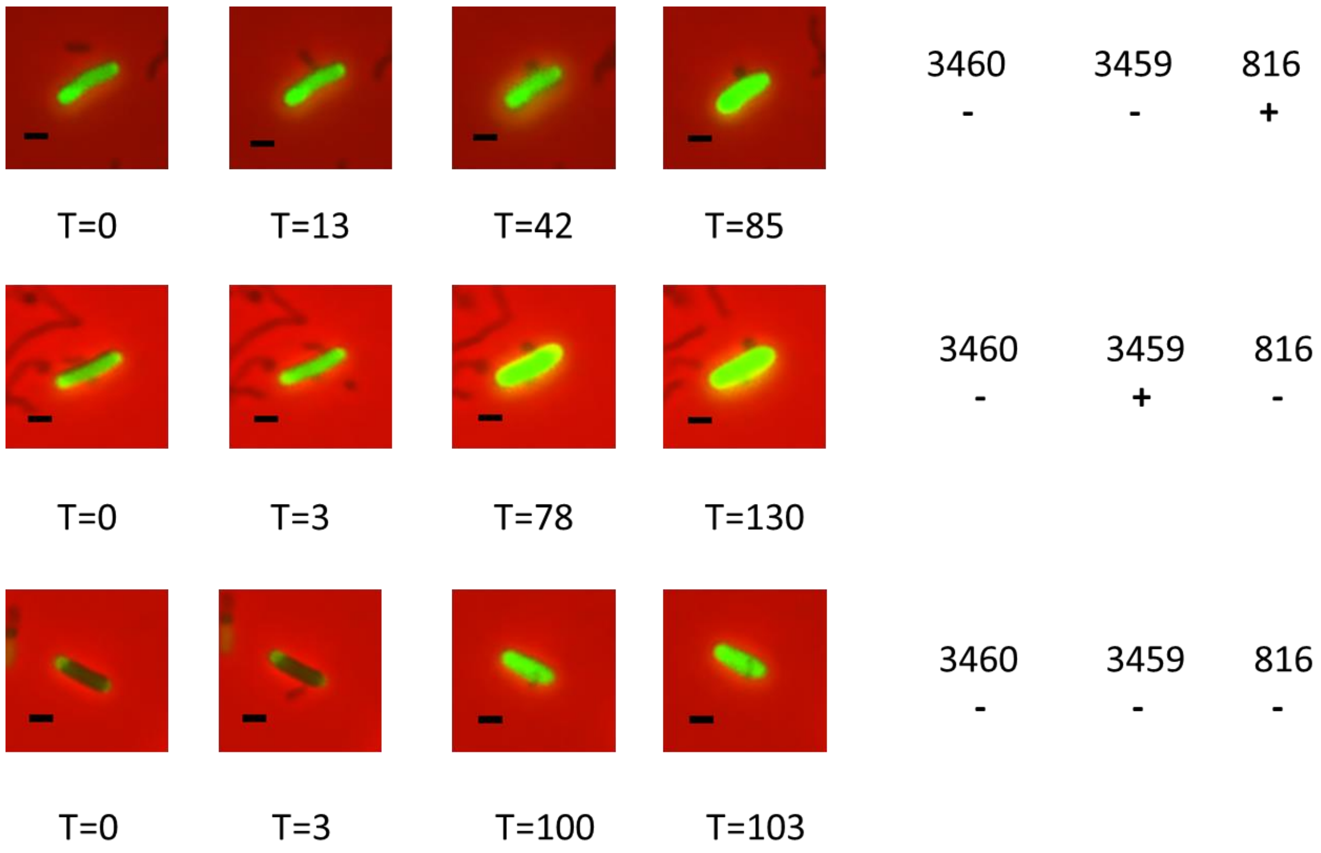

Supplementary Figure 6 | Combinatorial analysis of Bd0816:Bd3459:Bd3460 phenotypic relationships. Epifluorescence phase contrast microscopy of *Bdellovibrio* (small, phase dark, initially comma-shaped cells) attaching to *E. coli* prey cells which have their periplasm constitutively fluorescently labeled by a pMal::mCherry fusion. Deletion strains of *bd3460* that are wild-type for either *bd0816* or *bd3459* round up on contact with prey cells, but *Bdellovibrio* cells of a *bd3460* deletion strain also lacking both of these endopeptidase genes do not round up and can enter prey and grow host-dependently (-/-/-). In this strain, the absence of both *bd0816* and *bd3459* means that the prey is also not rounded up upon *Bdellovibrio* entry. Scale bars are 1μm and time since addition to prey (T) is indicated in minutes.

a

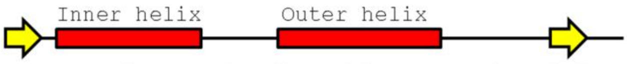


```
euk nGrTPLHlAarnGh--levVklLLeaGAdvnakdk
bac dGnTPLHlAaenGn--leiVklLLeaGAdvnaadl

 28 KSSKALNEAAEQGD--LAKVKNLVQKNKIDLNAQDE  61 (AR1)

 62 TGMTPLMNAAMGGN--LDIVKFLLSKKVNLELKNN   94 (AR2)

 95 GGETALAFAVTNDA--YDVAEELIKAGANVDIIVAGD 129(AR3)

130 EGDTLFMRAAQNN---KKTAESILAKNKSLINKANT  162(AR4)

163 LGETALFAVARYGT--PADIDFLIKKGADLKLKNK   195(AR5)

196 KGQTALDVAKEASN--QDTAKALSKKK*          220(AR6)
             [--turn--]
```

b

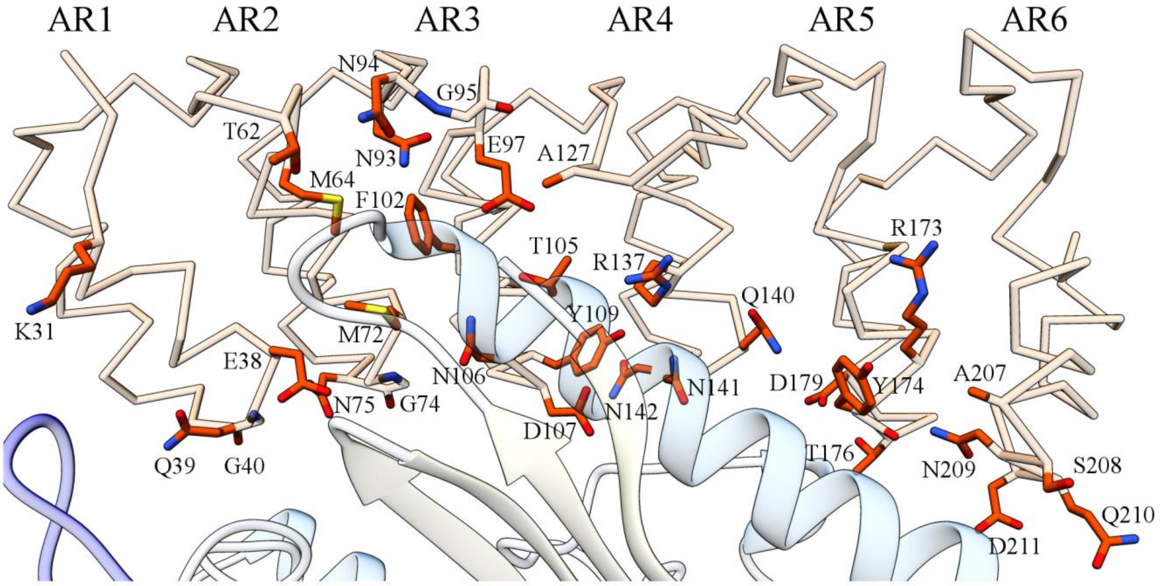

Supplementary Figure 7 | Ankyrin repeats of Bd3460. a, Eukaryotic (euk) and bacterial (bac) ankyrin consensus and fit to Bd3460 sequence repeats (consensus residues in Bd3460 boldtype, interacting residues red font). The interaction face is largely comprised of residues located at the α-α turn of the repeats. **b,** Interaction surface of Bd3460 (interacting residues in stick form) with Bd3459 transparent.

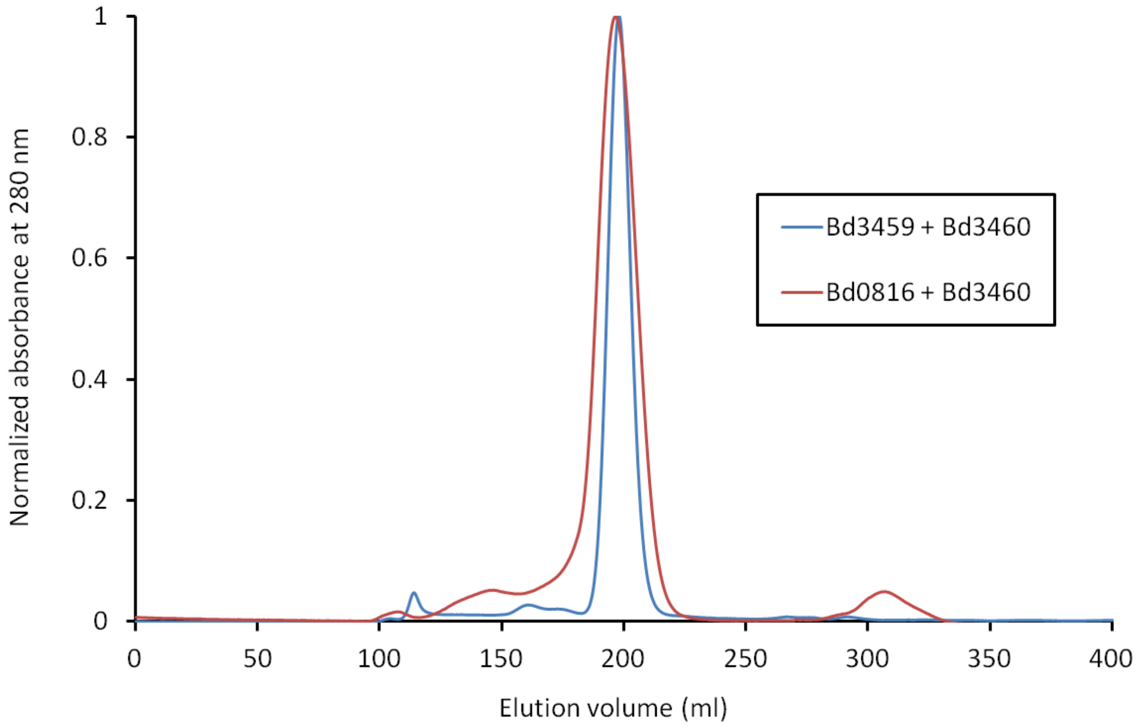

Supplementary Figure 8 | Analysis of endopeptidase:Bd3460 complexes by gel filtration. Protein complexes, Bd3459:Bd3460 and Bd0816:Bd3460, were over-expressed in *E. coli* and purified by nickel affinity chromatography prior to gel filtration analysis on a HiLoad Superdex 200 26/600 column (GE Healthcare). Approximately 20 mg of each protein complex was resolved on the chromatography medium and the absorbance at 280 nm, normalized to the maximum absorbance for each sample, was plotted against elution volume. The peaks in absorbance at 280 nm for both samples correspond to a molecular weight of approximately 72 kDa. Since the hypothetical molecular weights of Bd3459:Bd3460 and Bd0816:Bd3460 heterodimers are each approximately 69 kDa this indicates that each complex exists predominantly as a heterodimer in solution.

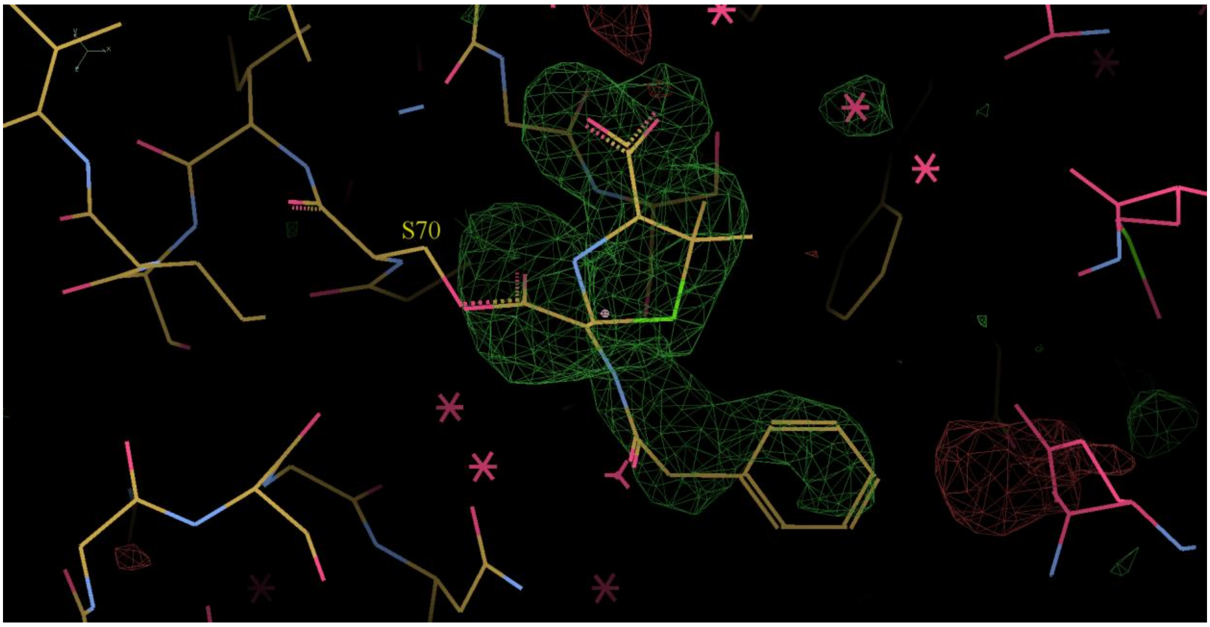

Supplementary Figure 9 | Bd3459:Bd3460 complex soaked with Penicillin G. Bd3459 (yellow) and Bd3460 (red) interface, showing that active site residue S70 was able to react with penicillin G (acyl adduct shown in stick form, original unmodeled positive F_o-F_c electron density contoured at 2.5σ coloured green). This implies that Bd3459 retains activity under complexation (although not active as an endopeptidase due to steric constraints imposed by Bd3460). Acylation of Bd3459 alters conformation of the nearby SXN motif and its flanking residue (AA 250-253).
